# Genetic Complexity of CC5 *Staphylococcus aureus* Isolates Associated with Sternal Bursitis in Chickens: Antimicrobial Resistance, Virulence, Plasmids, and Biofilm Formation

**DOI:** 10.3390/pathogens13060519

**Published:** 2024-06-20

**Authors:** Vanessa Silva, Jessica Ribeiro, Pedro Teixeira, Pedro Pinto, Madalena Vieira-Pinto, Patrícia Poeta, Manuela Caniça, Gilberto Igrejas

**Affiliations:** 1 LAQV-REQUIMTE, Department of Chemistry, NOVA School of Science and Technology, Universidade Nova de Lisboa, 2829-516 Caparica, Portugal; 2 Department of Genetics and Biotechnology, University of Trás-os-Montes and Alto Douro (UTAD), 5000-801 Vila Real, Portugal; 3 Functional Genomics and Proteomics Unit, University of Trás-os-Montes and Alto Douro (UTAD), 5000-801 Vila Real, Portugal; 4 Microbiology and Antibiotic Resistance Team (MicroART), Department of Veterinary Sciences, University of Trás-os-Montes and Alto Douro (UTAD), 5000-801 Vila Real, Portugal; 5 National Reference Laboratory of Antibiotic Resistances and Healthcare Associated Infections, Department of Infectious Diseases, National Institute of Health Dr. Ricardo Jorge, 1649-016 Lisbon, Portugal; 6 Department of Veterinary Sciences, University of Trás-os-Montes and Alto Douro (UTAD), 5000-801 Vila Real, Portugal; 7 Associate Laboratory for Animal and Veterinary Sciences (AL4AnimalS), 5000-801 Vila Real, Portugal; 8 CECAV—Veterinary and Animal Research Centre, University of Trás-os-Montes and Alto Douro, 5000-801 Vila Real, Portugal; 9 Centre for the Studies of Animal Science, Institute of Agrarian and Agri-Food Sciences and Technologies, University of Porto, 4051-401 Porto, Portugal

**Keywords:** *Staphylococcus aureus*, poultry, sternal bursitis, breast blisters, antimicrobial resistance, genetic lineages, CC5

## Abstract

Sternal bursitis, a common inflammatory condition in poultry, poses significant challenges to both animal welfare and public health. This study aimed to investigate the prevalence, antimicrobial resistance, and genetic characteristics of *Staphylococcus aureus* isolates associated with sternal bursitis in chickens. Ninety-eight samples were collected from affected chickens, and 24 *S. aureus* isolates were identified. Antimicrobial susceptibility testing revealed resistance to multiple agents, with a notable prevalence of aminoglycoside resistance genes. Whole genome sequencing elucidated the genetic diversity and virulence profiles of the isolates, highlighting the predominance of clonal complex 5 (CC5) strains. Additionally, biofilm formation assays demonstrated moderate biofilm production capacity among the isolates. These findings underscore the importance of vigilant monitoring and targeted interventions to mitigate the impact of sternal bursitis in poultry production systems.

## 1. Introduction

The prevalence of sternal bursitis, characterized by inflammation of the sternal bursa, is notably high across various production systems, indicating its significance in poultry health management [1,2]. The sternal bursa serves as a protective cushion against excessive wear, such as pressure from perches during resting periods. Excessive wear can trigger fluid accumulation within the bursa, leading to a local inflammatory response and the development of bursitis presternalis, commonly known as breast blisters [1]. Infectious sternal bursitis is a prevalent condition, yet it necessitates differentiation from traumatic sternal bursitis, characterized by fluid accumulation without an accompanying inflammatory exudate. Instances of purulent bursitis, where the sternal bursa becomes pus-filled, often involve infectious agents like *Staphylococcus* spp., *Streptococcus* spp., *Escherichia coli*, and *Mycoplasma* spp. [3]. Among these, *Staphylococcus aureus* predominates, contributing to acute septic bursitis, followed by *Streptococcus* species and other bacterial strains [4]. The condition of an enlarged sternal bursa is multifactorial, influenced by genetics, farm conditions, and management practices, leading to welfare concerns and economic losses in poultry farming [5]. Trauma or infection can inflame the bursa, resulting in fluid accumulation and the formation of fluid-filled blisters, with contributing factors including poor feathering, hard flooring, and leg weakness [6]. Scratches, hematomas, footpad dermatitis, breast blisters, and hock burn are common findings during post-mortem inspections, reflecting welfare conditions during rearing, transportation, and handling of broilers [7]. Despite the high prevalence of inflamed bursas, the risk of secondary bacterial infection remains relatively low if the integument remains intact [1].

Global expansion of the poultry industry has led to a significant rise in the incidence of zoonotic diseases, with staphylococci emerging as prominent bacterial pathogens in chickens [8]. Molecular epidemiology studies have delineated highly structured populations of *S. aureus*, characterized by clonal complexes (CCs) that exhibit genetic relatedness based on MLST loci [9]. Among these, CC5 and CC398 have been identified as livestock-associated lineages, frequently isolated from poultry, humans, and other hosts [10,11,12]. Of particular concern is CC5, which stands out as a predominant disease-causing lineage in chickens [8,13]. *S. aureus*, which is renowned for its global prevalence, poses a significant public health threat, causing a spectrum of infections ranging from uncomplicated skin infections to life-threatening invasive diseases [14]. The pervasive nature of antibiotic resistance further compounds the challenge, with *S. aureus* exhibiting resistance to multiple antibiotic classes [14]. Moreover, biofilm formation contributes to nonspecific antibiotic resistance, particularly in biofilm-associated infections [15]. The pathogenic versatility of *S. aureus* is underscored by its ability to cause a myriad of infections, including mild skin ailments, infective endocarditis, osteomyelitis, bacteremia, and lethal pneumonia [16]. Despite its pathogenicity, *S. aureus* exhibits remarkable adaptability, evolving resistance mechanisms against various antimicrobial agents commonly employed in treatment [15]. While zoonotic transmission of *S. aureus* is well-documented, elucidating the precise sources of *S. aureus* in human populations remains a subject of ongoing investigation [17]. Molecular epidemiological studies bridging animal and human reservoirs are pivotal for delineating the origins of *S. aureus* and MRSA strains, understanding their pathogenic attributes, and devising effective control measures. Additionally, determining the antimicrobial susceptibility profiles of *S. aureus* isolates is imperative for guiding targeted empirical therapy [17].

Understanding the genetic complexity of *S. aureus* isolates associated with sternal bursitis is crucial for effective management strategies. Next-generation sequencing (NGS) analysis offers a comprehensive approach to unraveling the antimicrobial resistance profiles, virulence factors, plasmid content, and biofilm formation capabilities of these isolates. By elucidating the genetic determinants contributing to the pathogenicity of *S. aureus* strains in sternal bursitis cases, targeted interventions can be developed to mitigate the impact of this prevalent poultry health issue. Thus, our objective is to utilize NGS analysis to investigate the genetic complexities of *S. aureus* isolates linked with sternal bursitis, aiming to elucidate their interconnected antimicrobial resistance, virulence profiles, plasmid dynamics, and biofilm formation capabilities within poultry production systems.

## 2. Materials and Methods

### 2.1. Sample Collection

Ninety-eight samples were collected at the slaughterhouse upon detection of bursitis in the chickens, ensuring that the specimens were obtained promptly following onset of the condition. The procedure for collecting pus from infected bursitis lesions in chickens was meticulously conducted to ensure the acquisition of high-quality samples. Initially, the area of the lesion was carefully prepared to minimize external contamination and ensure asepsis throughout the process. Firstly, the skin surface around the lesion was disinfected using 70% alcohol to eliminate superficial microorganisms and reduce the risk of contamination during collection. Following disinfection, the lesion site was carefully opened using sterile techniques to avoid the introduction of contaminating agents. A sterile swab was then used to collect the pus present in the lesion. The swab was gently inserted into the affected area, ensuring that all purulent material was collected uniformly and representatively. After collection, the swab was carefully removed and placed in transport medium to ensure preservation of the samples during transportation to a laboratory. Throughout the procedure, rigorous biosafety and aseptic measures were adopted to minimize the risk of cross-contamination and ensure the integrity of the collected samples. Each sample was taken from a different flock, and the samples originated from 11 different farms.

### 2.2. S. aureus Isolation

The swabs were introduced into tubes containing 5 mL of Brain Heart Infusion (BHI) broth supplemented with 6.5% NaCl and were then incubated at 37 °C for a duration of 24 h. Following the incubation period, the inoculum was plated onto Baird-Parker agar and Chromagar MRSA agar plates for the isolation of *S. aureus* and MRSA. From each plate, a maximum of three colonies displaying characteristics consistent with *S. aureus* but exhibiting morphological variations were selected. Species identification of *S. aureus* was conducted using biochemical assays (including catalase, DNase, and coagulase tests) as well as Bruker Biotyper MALDI-TOF MS analysis (Bruker Daltonics, Bremen, Germany)).

### 2.3. Assessment of Antimicrobial Susceptibility

Antimicrobial susceptibility testing was performed on all *S. aureus* isolates using the Kirby–Bauer disk diffusion method. Fourteen antimicrobial agents were tested, each at specific concentrations per disk (Oxoid, Basingstoke, UK): cefoxitin (30 µg), chloramphenicol (30 µg), ciprofloxacin (5 µg), clindamycin (2 µg), erythromycin (15 µg), fusidic acid (10 µg), gentamicin (10 µg), kanamycin (30 µg), linezolid (10 µg), mupirocin (200 µg), penicillin (1 U), tetracycline (30 µg), tobramycin (10 µg), and trimethoprim/sulfamethoxazole (1.25/23.75 µg). The interpretation of susceptibility results followed standards set by the European Committee on Antimicrobial Susceptibility Testing (EUCAST, 2024), with the exception of kanamycin, which adhered to the guidelines of the Clinical and Laboratory Standards Institute (CLSI, 2017). Quality control was ensured by including *S. aureus* strain ATCC 25923 in all assays.

### 2.4. Whole Genome Sequencing (WGS)

WGS was performed on a NextSeq 2000 Illumina platform. Read quality control and de novo assembly were performed using INNUca v4.2.2 (https://github.com/B-UMMI/INNUca accessed on 1 July 2023). All genomes were annotated with prokka v1.16.6. (doi:10.1093/bioinformatics/btu153). The average nucleotide identity (ANI) was calculated with fastANI v1.33 (https://github.com/ParBLiSS/FastANI accessed on 1 July 2023). WGS data were then analyzed to identify antibiotic resistance profiles using abricate v1.0.1 (https://github.com/tseemann/abricate#citation accessed on 1 July 2023) with CARD and NCBI AMRFinder databases. Multilocus Sequence Typing (MLST) was employed to categorize the isolates into clonal complexes and sequence types. Virulence genes were characterized with abritamr v1.0.14 (https://www.nature.com/articles/s41467-022-35713-4 accessed on 1 July 2023) and the pre-downloaded database VFDB in abricate.

### 2.5. Biofilm Formation

The investigation into biofilm formation was conducted using a microtiter assay method, following previously established procedures with slight adaptations [17]. Initially, two/three colonies from fresh staphylococci cultures were transferred to tubes containing 3 mL of Tryptic Soy Broth and incubated at 37 °C for 16 ± 1 h with continuous shaking at 150 rpm. Subsequently, a standardized suspension of staphylococci at 10^6^ cfu/mL was prepared, and 200 µL of this suspension was added to each well of a 96-well plate. *S. aureus* ATCC^®^ 25923 served as the positive control in all plates, while uninoculated TSB acted as the negative control. The plates were then incubated at 37 °C for 24 h under static conditions. Each experiment was conducted with seven technical replicates and performed in triplicate.

#### Biofilm Biomass Quantification

Biofilm biomass quantification was performed using the Crystal Violet (CV) Staining method, as previously outlined by Peeters et al. (2008), with some modifications [18]. Following the incubation period, the wells of the plate were washed twice with 200 µL of distilled water to eliminate non-adherent bacterial cells, after which the plates were allowed to air-dry at room temperature for 2 h. Subsequently, the biofilm cells were fixed with 100 µL of methanol and incubated for 15 min at room temperature. After removal of the methanol, the plates were left to dry in a laminar flow cabinet for 10 min. The attached biofilm cells were then stained with 100 µL of 1% (*v*/*v*) Crystal Violet (CV) solution for 10 min at room temperature. Excess dye was eliminated by washing the plates with distilled water, and Crystal-Violet-bound cells were solubilized with 33% (*v*/*v*) acetic acid. Absorbance was measured at 570 nm using a microplate reader BioTek ELx808U. To standardize the results, biofilm formation of each isolate was normalized based on the results obtained from the positive control strain ATCC^®^ 25923.

## 3. Results and Discussion

### 3.1. Prevalence and Antimicrobial Resistance

**Figure 1:**
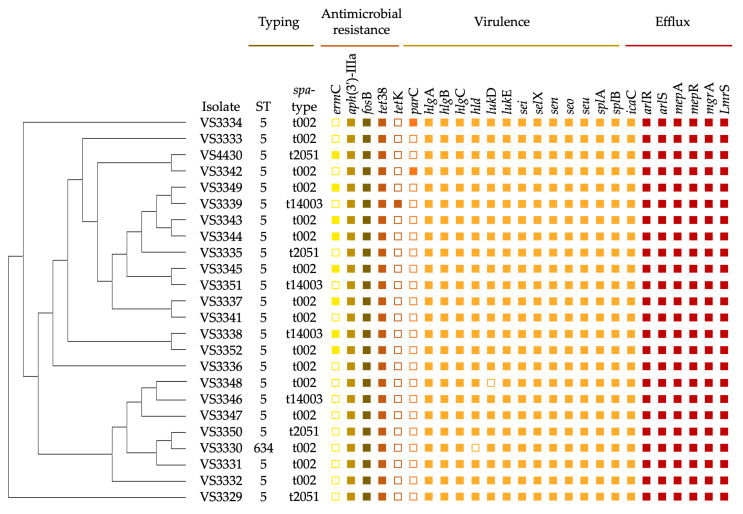
Phylogenetic tree showing 24 *S. aureus* isolates analyzed via NGS, showcasing MLST, *spa*-type, resistance gene, and virulence factors profiles. The unrooted maximum likelihood tree, ordered and constructed using RAxML, was generated based on ‘Core Genome SNPs. Filled squares indicate the presence of the corresponding gene, while empty squares indicate the absence of the gene.

*S. aureus* is recognized as a prevalent opportunistic pathogen in poultry, capable of causing a wide range of infections including dermatitis, arthritis, and most relevantly for this study, sternal bursitis [3,4,19,20]. In our study, among 98 samples recovered from chicken sternal bursitis, 24 (24.5%) *S. aureus* isolates were isolated. However, no MRSA was detected. *S. aureus* infection in poultry poses a significant concern in both the poultry industry and public health. Chicken sternal bursitis (CSB), also known as breast blisters, can lead to significant animal welfare issues, and reduced production and meat quality, as well as food safety concerns due to potential bacterial contamination [21]. Nevertheless, recent literature specifically on this topic is very scarce. Indeed, as far as we are aware, this is the first study reporting the prevalence, antimicrobial resistance, and genetic lineages of *S. aureus* causing sternal bursitis in chickens. Since there are no available studies to compare the frequency of *S. aureus* associated with CSB, we examined the occurrence of *S. aureus* in other poultry lesions as a reference point. Several studies have shown an *S. aureus* prevalence lower than the one obtained in this study. Marcon et al. analyzed 60 samples collected from chickens with arthritis, and bacterial growth for *S. aureus* was detected in 3.3% of samples to a mild degree and in 10% to a severe degree [22]. While in a study by El-Tawab et al., the prevalence of *S. aureus* in poultry arthritis was 19% [23]. Another study reported a prevalence of 11.5% of *S. aureus* in poultry bacterial chondronecrosis and osteomyelitis [24]. In a study by Heidemann Olsen et al. conducted with chicken pododermatitis, 15 (14%) of the 111 samples were positive for *S. aureus* [25]. Nevertheless, other studies have shown a higher prevalence of *S. aureus* causing infections in chickens. Nazia et al. described the frequency of septic arthritis caused by *S. aureus*, which was up to 81% [26]. Regarding antimicrobial resistance, all isolates were resistant to aminoglycosides and all carried the *aph*(3′)-IIIa gene (Figure 1). This gene is a plasmid-encoded minocyclitol-3′-phosphotransferase and mediates resistance to amikacin, neomycin, and kanamycin in *S. aureus* [27,28]. The *aph*(3′)-IIIa gene rate in this study was very high, which contrasts with the lower frequencies of aminoglycoside resistance observed in other studies on *S. aureus* from poultry [10,29,30]. In contrast, most studies conducted with poultry and livestock in general have reported a high frequency of tetracycline resistance; this trend was not observed in our study [10,16,29,30,31]. In fact, tetracycline holds a prominent position, as evidenced by sales data on veterinary antimicrobial agents in Portugal from a European Medicines Agency (EMA) report [32]. The report highlights tetracyclines as the most frequently sold antimicrobials, followed by penicillins, macrolides, lincosamides, fluoroquinolones, and aminoglycosides. In our study, only one isolate showed phenotypic resistance to tetracycline and carried the *tet*K gene. All isolates carried the *tet*38 gene, which confers resistance to tetracycline and can increase resistance up to 32-fold when overexpressed. However, expression of this gene is significantly heightened under infection conditions that trigger its up-regulation [33]. Additionally, the Tet38 efflux pump is regulated by *mgr*A, which is an indirect negative regulator of *tet*38 gene expression. Since *mgr*A was present in all isolates, it may explain why tetracycline resistance was not observed in 23 isolates [34]. Eight isolates showed resistance to erythromycin and clindamycin and carried the *erm*C gene. This prevalence of *erm*C aligns with previous research, such as studies conducted in healthy poultry populations in Portugal, China, and Egypt, where *erm*C was also identified as the most prevalent gene [10,35,36,37]. Nevertheless, other studies have reported *erm*C as the least prevalent among the *erm* genes in healthy poultry populations [38]. Resistance to ciprofloxacin was detected in two isolates and both harbored the *par*C gene. A point mutation in the *par*C gene of *S. aureus* leads to resistance against fluoroquinolones [39]. The gene *fos*B, conferring resistance to fosfomycin, was also detected in all *S. aureus* isolates. The heightened expression of a chromosomally located *fos*B gene, responsible for encoding an enzyme that inactivates fosfomycin, has been shown in several *S. aureus* isolates. However, its presence alone does not consistently correlate with fosfomycin resistance [40]. Nevertheless, it has been shown that certain clonal lineages of *S. aureus* possess an inherent chromosomal *fos*B gene, as is the case with ST5, and can potentially lead to fosfomycin resistance through increased expression of this gene [40,41]. The identification of antimicrobial resistance genes in *S. aureus* strains isolated from poultry has significant implications for both veterinary and human medicine. The presence of resistance genes such as *aph*(3′)-IIIa, which mediates resistance to aminoglycosides, and *erm*C, which confers resistance to macrolides and lincosamides, suggests limited treatment options for infections caused by these strains. In veterinary settings, this can lead to difficulties in managing infections in poultry, necessitating the use of alternative or higher doses of antimicrobials, which may not always be feasible or effective [27,28,42]. In veterinary medicine, the presence of these resistance genes indicates a need for stringent antimicrobial stewardship to prevent the escalation of resistant strains. For example, tetracycline resistance mediated by the *tet*K and *tet*38 genes could compromise the efficacy of this commonly used antibiotic in poultry farming. Monitoring the use of these antibiotics and implementing alternative treatments when resistance is detected are crucial steps in managing animal health effectively [43,44]. The zoonotic potential of these antimicrobial-resistant strains poses a significant public health risk. Human infections with poultry-associated S. aureus strains, especially those carrying resistance genes, can range from skin infections to more severe conditions like bacteremia and endocarditis. The potential for these strains to enter the human food chain highlights the importance of rigorous food safety measures and monitoring systems to detect and control the spread of resistant bacteria from animals to humans [45,46].

### 3.2. Molecular Typing

All isolates were typed by MLST and *spa*-typing, and 23 of the 24 isolates were Sequence Type 5 (ST5) and one isolate was ST634. Nevertheless, all isolates were grouped in CC5. In fact, ST634 differs from ST5 by a single point mutation on the *arc*C locus. Isolates were assigned to three different *spa*-types: t002 (*n* = 16), t2051 (*n* = 4), and t14003 (*n* = 4). ST5 has been widely detected in poultry populations across several countries [47,48,49]. *S. aureus* strains from poultry primarily belong to ST9, ST398, and ST5, often exhibit distinct virulence gene patterns [48]. Additionally, CC5, a widespread clonal complex, encompasses both community-associated and healthcare-associated methicillin-resistant *S. aureus* (MRSA) strains [44]. Notably, CC5 has been identified as an animal-adapted clone, recovered from various livestock species, such as poultry, pigs, and cattle, as well as companion animals [50,51]. Particularly, the *spa*-type t002 of ST5 is considered an endemic strain in livestock, and is believed to have transitioned from a nosocomial strain to livestock and subsequently become involved in human diseases, akin to the livestock-associated strain ST398 [52,53,54]. This lineage, represented by ST5-t002, has been detected in both poultry and humans [49,55]. A noteworthy observation is the host jump of CC5 strains, particularly ST5, to poultry populations, where they frequently contribute to disease outbreaks [52]. Studies by Lowder et al. have suggested a recent human-to-poultry host jump of the ST105-t002-II lineage, possibly facilitated by close contact between human and poultry populations. This underscores the adaptability of ST5 to various hosts, with implications for both community- and hospital-associated MRSA infections [52]. Furthermore, the remarkable capacity of the ST5 lineage to acquire mobile genetic elements enhances its ability to adapt to new hosts, such as poultry and pigs [54]. Regarding *S. aureus* t2051, it has primarily been associated with human populations [56,57]. ST5 strains of *S. aureus* exhibit host-specific adaptations, with those infecting chickens often differing in virulence factors and resistance profiles compared to human-adapted strains. Despite these differences, the potential for zoonotic transmission remains significant, particularly in environments with close human–poultry interactions. Enhanced biosecurity measures and surveillance are essential to monitor and control the spread of these strains [10,12,44,51].

### 3.3. Virulence Factors

All isolates carried genes coding for α-, β-, and γ-hemolysin, namely *hla*, *hlb*, and *hlg*C, respectively, and 22 of 24 isolates also carried the *hld* gene (δ-hemolysin). These results come as no surprise, as the *hla* gene is present in the vast majority of *S. aureus* strains (95%) irrespective of their methicillin-resistance status. This presence does not demonstrate a distinct distribution among *S. aureus* clones or a higher prevalence in specific geographic regions [58]. Moreover, studies have shown that synthesis of Hlb in *S. aureus*, which is encoded by the *hlb* gene, is associated with a loss of Sa3int phages during the transmission of bacteria from humans to livestock. This implies that our isolates are not of human origin [59]. Indeed, none of our isolates carried the *scn* gene, which typically signifies the presence of the immune evasion cluster (IEC) system. The presence of IEC genes often indicates a potential human origin [60]. It has been shown that the lack of IEC genes in *S. aureus* ST5 strains mirrors the observations seen in poultry-adapted ST5 strains, where genes specific to humans were lost and replaced by those encoding avian-specific factors after the transition from humans to poultry [54]. All isolates carried the genes *luk*E and *luk*D, which are responsible for encoding leukocidin ED toxin. These two genes, *luk*E and *luk*D, are known for their high expression levels and code for pore-forming leukotoxins. Leukocidin ED (LukED) is a potent pore-forming toxin produced by *S. aureus*, and it functions by lysing host cells, thereby enhancing bacterial virulence [61]. Based on antigenic diversity, researchers have identified over 20 different staphylococcal enterotoxins (SEs), ranging from SEA to SElV [62,63]. These enterotoxins possess the ability to stimulate large populations of T cells, resulting in an uncontrolled activation of the immune system [64]. The *S. aureus* strains identified in this study, which were responsible for sternal bursitis, may have initially established themselves as commensal organisms or colonized the feathers and mucous membranes of the chickens. With the chickens’ immune defenses compromised, these bacteria seized the opportunity to become opportunistic pathogens, leading to sternal bursitis. Consequently, our findings suggest that these isolates could be persistently colonizing the chicken population. In cases where sternal bursitis is absent, these chickens would likely proceed to slaughter for human consumption. Notably, all of our isolates carried five enterotoxin genes (*sei*, *sel*X, *sen*, *seo*, and *seu*), indicating a potential risk to consumers. Staphylococcal food poisoning is primarily caused by SEs. Upon ingestion of food contaminated with these toxins, individuals may experience acute gastroenteritis, diarrhea, vomiting, and abdominal pain [62,63]. All isolates also carried serine protease genes (*spl*A and *spl*B), one intercellular adhesin gene (*ica*C), and an antiphagocytic fibrinogen gene (*sem*). Six serine protease-like proteins, known as SplA to SplF, are expressed by the majority of clinical *S. aureus* isolates. However, studies have demonstrated that the deletion of *spl*ABCDEF did not result in a significant reduction in virulence or overall virulence attenuation [65,66]. All isolates carried the multidrug resistance efflux pumps *arl*R, *arl*S, *nor*A, *mep*A, *mep*R, *mgr*A, and *lmr*S. All isolates harbored the *lmr*S, *nor*A, and *mep*A genes, with two of them also containing the *qac*G gene, which may also confer resistance to biocides. Chromosomally encoded efflux genes like *mep*A, *nor*A, and *lmr*S have been noted for their role in conferring multidrug and biocide resistance to bacteria, aiding in their survival under harsh conditions [67]. On the other hand, plasmid-mediated genes such as *qac*A/B encode proteins belonging to a major facilitator superfamily, while genes like *qac*G encode members of a small multidrug resistance family, which serve as the primary proteins for multidrug resistance efflux pumps [67]. In animal husbandry, biocides like QACs find widespread usage [68]. These compounds serve as antiseptics for treating minor skin injuries in animals and as disinfectants for surfaces and workers’ hands at farms, slaughterhouses, and food processing facilities, ensuring necessary standards of hygiene [69]. Presence of the *qac*G gene may confer a beneficial trait to bacteria, facilitating the colonization of animals, as well as their environment and humans. This could potentially contribute to the selection and dissemination of *S. aureus* ST5.

### 3.4. Plasmids

*S. aureus* is recognized as a repository of genes conferring antibiotic resistance, with insertion sequences or transposons facilitating gene dissemination. It is crucial to conduct vigilant monitoring to detect strains capable of transferring antibiotic resistance. The presence of plasmids was investigated using PlasmidFinder, and five plasmids were detected: repUS20, repUS5, rep10, rep7a, and rep21. Plasmids were present in 22 of the 24 isolates with repUS5 (*n* = 20) being the most frequent followed by repUS20 (*n* = 16) and rep10 (*n* = 6) (Supplementary Table S1). Plasmid replicons repUS5 and repUS20 have been reported among livestock-associated *S. aureus* isolates, particularly those associated with poultry and *S. aureus* CC5 [52,70,71]. In addition, in samples from poultry, the presence of repUS5 has previously been identified in *S. aureus* linked to the transfer of antimicrobial resistance genes among staphylococci [70]. Plasmid rep10 exhibits close similarity to plasmid pDLK1 documented in Kuntová et al.’s study [72]. In that study, pDLK1 was found in CC5 strains and correlated with erythromycin resistance, carrying the *erm*C gene, which is a finding consistent with our own results. In our study, all isolates harboring rep10 also contained the *erm*C gene. Plasmids rep21 and rep7a were identified in only one isolate each (VS3337 and VS3339, respectively) and have previously been documented in livestock-associated *S. aureus* strains, particularly of the ST398 lineage [73,74]. Additionally, rep7a has been reported to harbor the *tet*K gene, which is a finding consistent with our detection of the *tet*K gene in our VS3339 isolate [74]. Plasmids play a crucial role in the horizontal transfer of antimicrobial resistance genes among bacteria, which can facilitate the spread of resistance within and between different bacterial populations. In our study, the detection of plasmids such as repUS5 and rep10 in *S. aureus* isolates underscores their role in harboring resistance genes like *erm*C and contributing to erythromycin resistance. These plasmids enhance bacteria’s ability to acquire and disseminate resistance genes, complicating treatment strategies and necessitating continuous surveillance and control measures.

### 3.5. Biofilm Formation

**Figure 2:**
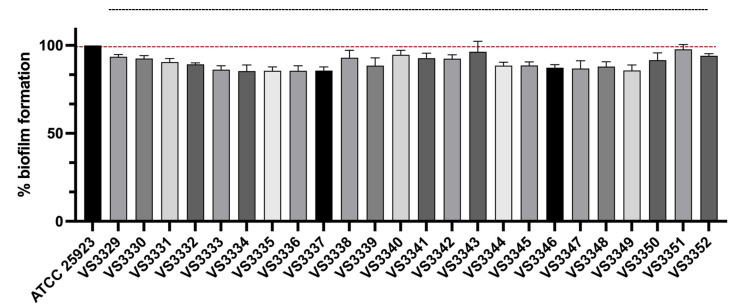
Percentage of biofilm formation of 24 *S. aureus* strains isolated from chicken bursitis. Data are presented as mean ± standard deviation for seven independent replicates. To standardize the results, biofilm formation of each isolate was normalized based on the results obtained from the positive control strain ATCC^®^ 25923.

In the context of sternal bursitis in chickens, *S. aureus* is known to exhibit various virulence factors that contribute to its pathogenicity. Among these, biofilm formation is a critical factor that enhances bacterial persistence and resistance to treatment. Biofilms provide a protective environment for bacterial colonies, shielding them from the host’s immune response and antimicrobial agents. Given its significant impact on the chronicity and treatment resistance of infections, biofilm formation was selected as a focal point for our analysis. All isolates had the ability to form a biofilm. Biofilm formation results are shown in Figure 2 and were obtained by normalizing the absorbance value of each strain with the absorbance value of the control strain *S. aureus* ATCC25923. However, none of the isolates were classified as high-biofilm formers when compared to the control strain. The moderate biofilm formation capacity of all isolates may be due to the absence of *ica* and MSCRAMM (microbial surface components recognizing adhesive matrix molecules) genes. MSCRAMM are adhesin proteins that play a key role in enabling bacteria to attach to host tissue initially, which is a critical step in the process of infection establishment, and are encoded by the *bap*, *cna*, *ebps*, *eno*, *fnb*A, *fnb*B, fib, *clf*A, and *clf*B genes. Indeed, all isolates carried the *ica*C gene but lacked the other ica genes (*ica*A, *ica*B, and *ica*D). The *ica* operon, present in *S. aureus*, regulates polysaccharide intercellular adhesin (PIA) synthesis. Deletion of this operon prevents PIA production and biofilm formation [75,76]. The formation of biofilms is strongly linked to the presence of *ica* genes. Research indicates that *S. aureus* biofilms expressing *ica* genes tend to develop thicker and more densely structured biofilms compared to *ica*-negative isolates [77,78]. A prior study conducted by our team on S. aureus isolated from quails revealed that these isolates produced significantly greater biofilm biomass compared to a control strain [79]. Other studies, conducted with *S. aureus* isolated from poultry, mainly from poultry meat, have shown that most *S. aureus* from poultry are strong biofilm producers with a few being moderate producers [80,81,82,83].

The zoonotic potential of *S. aureus*, particularly the CC5 strains, is a significant concern due to the potential transmission between poultry and humans. Studies have demonstrated that CC5 is a predominant lineage in poultry, exhibiting distinct virulence gene patterns and adaptability to various hosts, including humans, livestock, and companion animals. This adaptability is facilitated by the acquisition of mobile genetic elements, which enhance the ability of CC5 strains to colonize and infect new hosts [52,84,85,86,87]. Transmission of *S. aureus* from poultry to humans can occur through direct contact with infected animals, consumption of contaminated poultry products, or via the environment in farms and slaughterhouses. The host jump of CC5 strains to poultry populations, where they frequently cause disease outbreaks, underscores the importance of monitoring and controlling these transmissions [8,12,52]. Human infections with poultry-associated *S. aureus*, particularly those caused by antimicrobial-resistant strains, pose significant public health risks [54,58]. These infections can range from skin infections to more severe conditions like bacteremia and endocarditis [14,55]. The presence of enterotoxin genes in poultry-associated *S. aureus* isolates also raises concerns about foodborne illnesses [13,57]. To mitigate the transmission and impact of *S. aureus*, particularly CC5 strains, a combination of biosecurity measures, antimicrobial stewardship, and surveillance is essential [10,12]. On poultry farms, implementing strict hygiene practices, monitoring flock health, and reducing the use of antibiotics can help control the spread of these pathogens [10]. In human healthcare settings, it is crucial to monitor and manage infections caused by poultry-associated *S. aureus*, ensuring appropriate treatment and infection control measures are in place [44]. Given the identified resistance genes and their potential for horizontal transfer, it is essential to implement comprehensive antimicrobial stewardship programs in both veterinary and human healthcare settings [19]. This includes monitoring antibiotic use, promoting the judicious use of antimicrobials, and encouraging the development and use of alternative treatments. Enhanced biosecurity measures, such as improving hygiene practices and monitoring flock health on poultry farms, are also critical in controlling the spread of these pathogens [19,21,22,80].

## 4. Conclusions

Our study sheds light on the genetic characteristics of *S. aureus* isolates associated with sternal bursitis in poultry. The prevalence of *S. aureus* in these samples underscores its significance in poultry health, with antimicrobial resistance genes posing concerns for treatment efficacy. The predominance of CC5 highlights its adaptability and potential transmission between poultry and humans. Virulence gene profiles suggest the pathogenic potential of these isolates, with the presence of enterotoxin genes posing food safety risks. Plasmid dynamics indicate the potential for horizontal gene transfer, emphasizing the need for vigilant monitoring. This can be achieved through techniques such as plasmid profiling and the surveillance of antimicrobial resistance patterns in bacterial populations. Overall, understanding the genetic complexity of *S. aureus* in sternal bursitis is crucial for implementing effective control measures and safeguarding both poultry welfare and public health.

## Data Availability

The original contributions presented in the study are included in the article/Supplementary Material; further inquiries can be directed to the corresponding author/s.

## Supplementary Materials

The following supporting information can be downloaded at https://www.mdpi.com/article/10.3390/pathogens13060519/s1: Table S1: Plasmids of *S. aureus* isolated from chicken sternal bursitis.
