# Supplementary material for: Genetic Complexity of CC5 Staphylococcus aureus Isolates Associated with Sternal Bursitis in Chickens: Antimicrobial Resistance, Virulence, Plasmids, and Biofilm Formation
Source: Pathogens. 2024 Jun 20;13(6):519. doi: 10.3390/pathogens13060519 (PMC11206601; doi:10.3390/pathogens13060519)
Supplement: Supplementary file 1 [file pathogens-13-00519-s001.zip › pathogens-3022197-supplementary.pdf]

Table S1. Plasmids of *S. aureus* isolated from chicken sternal bursitis.

Strain	Plasmid	Identity	Query / Template Lenght	Contig	Position in Contig	Closest Plasmid	Accession number
VS3329	repUS5	100	1575 / 1575	NODE_50_length_9311_cov_610.524417	5798..7372	BJL6515670(pAVX)	CP017805
	repUS20	100	1284 / 1284	NODE_56_length_3685_cov_688.934986	1570..2853	rep(pAVX)	CP001784
VS3330	repUS5	100	1575 / 1575	NODE_127_length_2450_cov_447.783299	155..1729	BJL6515670(pAVX)	CP017805
VS3331	repUS5	100	1575 / 1575	NODE_37_length_13962_cov_585.866973	10449..12023	BJL6515670(pAVX)	CP017805
	repUS20	100	1284 / 1284	NODE_37_length_13962_cov_585.866973	1856..3139	rep(pAVX)	CP001784
VS3332	repUS5	100	1575 / 1575	NODE_31_length_9273_cov_376.221632	5760..7334	BJL6515670(pAVX)	CP017805
	repUS20	100	1284 / 1284	NODE_36_length_3685_cov_464.890358	833..2116	rep(pAVX)	CP001784
VS3333	repUS5	100	1575 / 1575	NODE_70_length_6798_cov_672.042266	1824..3398	BJL6515670(pAVX)	CP017805
VS3334	repUS5	100	1575 / 1575	NODE_43_length_9273_cov_620.092428	5760..7334	BJL6515670(pAVX)	CP017805
	repUS20	100	1284 / 1284	NODE_50_length_3685_cov_736.466667	833..2116	rep(pAVX)	CP001784
VS3335	repUS5	100	1575 / 1575	NODE_40_length_10252_cov_569.858782	6530..8104	BJL6515670(pAVX)	CP017805
	repUS20	100	1284 / 1284	NODE_47_length_3685_cov_646.365565	833..2116	rep(pAVX)	CP001784
VS3336	repUS5	100	1575 / 1575	NODE_40_length_21253_cov_515.960279	1940..3514	BJL6515670(pAVX)	CP017805
	repUS5	100	1575 / 1575	NODE_40_length_21253_cov_515.960279	17740..19314	BJL6515670(pAVX)	CP017805
	repUS20	100	1284 / 1284	NODE_59_length_2900_cov_535.028471	48..1331	rep(pAVX)	CP001784
VS3337	rep21	98,86	1050 / 1050	NODE_40_length_3103_cov_2228.119423	699..1748	rep(pS0385)	AM990995
	repUS5	100	1575 / 1575	NODE_35_length_9933_cov_391.183921	1885..3459	BJL6515670(pAVX)	CP017805
	repUS20	100	1284 / 1284	NODE_37_length_3688_cov_479.455822	836..2119	rep(pAVX)	CP001784
	rep10	100	477 / 477	NODE_42_length_2417_cov_1854.096528	1731..2207	repL(pDLK1)	GU562624
VS3338	rep10	100	477 / 477	NODE_31_length_2462_cov_1342.951807	1776..2252	repL(pDLK1)	GU562624
	rep7a	100	750/750	NODE_94_length_4901_cov_811.888155	3934..4683	repC(pS0385p1	AM990993
VS3339	repUS5	100	1575 / 1575	NODE_75_length_9933_cov_282.626852	1885..3459	BJL6515670(pAVX)	CP017805
	repUS20	100	1284 / 1284	NODE_99_length_3974_cov_463.228119	836..2119	rep(pAVX)	CP001784

VS3340	No hits found						
VS3341	repUS5	100	1575 / 1575	NODE_33_length_9933_cov_462.256808	1885..3459	BJL6515670(pAVX)	CP017805
	repUS20	100	1284 / 1284	NODE_36_length_3688_cov_571.038260	836..2119	rep(pAVX)	CP001784
VS3342	repUS5	100	1575 / 1575	NODE_67_length_6798_cov_714.380543	1824..3398	BJL6515670(pAVX)	CP017805
VS3343	rep10	100	477 / 477	NODE_75_length_2462_cov_850.403822	347..823	repL(pDLK1)	GU562624
VS3344	repUS5	100	1575 / 1575	NODE_47_length_9933_cov_258.208851	1885..3459	BJL6515670(pAVX)	CP017805
	rep10	100	477 / 477	NODE_56_length_2462_cov_923.571666	331..807	repL(pDLK1)	GU562624
	repUS20	100	1284 / 1284	NODE_48_length_3974_cov_403.795611	836..2119	rep(pAVX)	CP001784
VS3345	repUS20	100	1284 / 1284	NODE_46_length_2900_cov_455.979965	48..1331	rep(pAVX)	CP001784
	repUS5	100	1575 / 1575	NODE_34_length_21115_cov_433.674834	17671..19245	BJL6515670(pAVX)	CP017805
	repUS5	100	1575 / 1575	NODE_34_length_21115_cov_433.674834	1871..3445	BJL6515670(pAVX)	CP017805
	rep10	100	477 / 477	NODE_45_length_2938_cov_1771.235865	1717..2193	repL(pDLK1)	GU562624
VS3346	repUS5	100	1575 / 1575	NODE_34_length_9933_cov_441.055867	1885..3459	BJL6515670(pAVX)	CP017805
	repUS20	100	1284 / 1284	NODE_36_length_3974_cov_667.305690	836..2119	rep(pAVX)	CP001784
VS3347	repUS5	100	1575 / 1575	NODE_37_length_13676_cov_526.825563	1940..3514	BJL6515670(pAVX)	CP017805
	repUS20	100	1284 / 1284	NODE_37_length_13676_cov_526.825563	10824..12107	rep(pAVX)	CP001784
VS3348	repUS5	100	1575 / 1575	NODE_165_length_3162_cov_276.051175	1385..2959	BJL6515670(pAVX)	CP017805
VS3349	repUS20	100	1284 / 1284	NODE_49_length_3685_cov_543.536639	833..2116	rep(pAVX)	CP001784
	rep10	100	477 / 477	NODE_45_length_5023_cov_2327.078704	4116..4592	repL(pDLK1)	GU562624
	rep10	100	477 / 477	NODE_45_length_5023_cov_2327.078704	432..908	repL(pDLK1)	GU562624
	repUS5	100	1575 / 1575	NODE_43_length_9273_cov_451.112823	5760..7334	BJL6515670(pAVX)	CP017805
VS3350	repUS5	100	1575 / 1575	NODE_26_length_9933_cov_490.907189	1885..3459	BJL6515670(pAVX)	CP017805
	repUS20	100	1284 / 1284	NODE_27_length_3974_cov_618.383261	836..2119	rep(pAVX)	CP001784
VS3351	No hits found						
VS3352	repUS20	100	1284 / 1284	NODE_49_length_6826_cov_861.781421	833..2116	rep(pAVX)	CP001784
	repUS5	100	1575 / 1575	NODE_51_length_4946_cov_715.747291	1549..3123	BJL6515670(pAVX)	CP017805
